# Periplocin and cardiac glycosides suppress the unfolded protein response

**DOI:** 10.1038/s41598-021-89074-x

**Published:** 2021-05-04

**Authors:** Muneshige Tokugawa, Yasumichi Inoue, Kan’ichiro Ishiuchi, Chisane Kujirai, Michiyo Matsuno, Masaki Ri, Yuka Itoh, Chiharu Miyajima, Daisuke Morishita, Nobumichi Ohoka, Shinsuke Iida, Hajime Mizukami, Toshiaki Makino, Hidetoshi Hayashi

**Affiliations:** 1 Department of Cell Signaling, Graduate School of Pharmaceutical Sciences, Nagoya City University, Nagoya, 467-8603 Japan; 2 Department of Innovative Therapeutic Sciences, Cooperative Major in Nanopharmaceutical Sciences, Graduate School of Pharmaceutical Sciences, Nagoya City University, Nagoya, 467-8603 Japan; 3 Department of Pharmacognosy, Graduate School of Pharmaceutical Sciences, Nagoya City University, Nagoya, 467-8603 Japan; 4 The Kochi Prefectural Makino Botanical Garden, Kochi, 781-8125 Japan; 5 Department of Hematology and Oncology, Graduate School of Medical Sciences, Nagoya City University, Nagoya, 467-8601 Japan; 6 Chordia Therapeutics Inc., Kanagawa, 251-0012 Japan; 7 Division of Molecular Target and Gene Therapy Products, National Institute of Health Sciences, Kawasaki, 210-9501 Japan

**Keywords:** Molecular medicine, Cell signalling

## Abstract

The unfolded protein response (UPR) controls protein homeostasis through transcriptional and translational regulation. However, dysregulated UPR signaling has been associated with the pathogenesis of many human diseases. Therefore, the compounds modulating UPR may provide molecular insights for these pathologies in the context of UPR. Here, we screened small-molecule compounds that suppress UPR, using a library of Myanmar wild plant extracts. The screening system to track *X-box binding protein 1* (*XBP1*) splicing activity revealed that the ethanol extract of the *Periploca calophylla* stem inhibited the inositol-requiring enzyme 1 (IRE1)-XBP1 pathway. We isolated and identified periplocin as a potent inhibitor of the IRE1-XBP1 axis. Periplocin also suppressed other UPR axes, protein kinase R-like endoplasmic reticulum kinase (PERK), and activating transcription factor 6 (ATF6). Examining the structure–activity relationship of periplocin revealed that cardiac glycosides also inhibited UPR. Moreover, periplocin suppressed the constitutive activation of XBP1 and exerted cytotoxic effects in the human multiple myeloma cell lines, AMO1 and RPMI8226. These results reveal a novel suppressive effect of periplocin or the other cardiac glycosides on UPR regulation, suggesting that these compounds will contribute to our understanding of the pathological or physiological importance of UPR.

## Introduction

The endoplasmic reticulum (ER) is the major organelle that mediates folding proteins, such as membrane or secretory proteins. Peptide chains newly synthesized from ribosomes are organized to their proper conformations in the ER. These peptides are then transferred to the Golgi apparatus for further post-translational modifications^1^. However, an aberrant subcellular milieu, including nutrient deprivation, hypoxia, the hypersecretion of proteins, or the repressed function of the ER, which causes ER stress, resulted in the excessive accumulation of misfolded proteins in the ER lumen^2,3^.

To cope with ER stress, a subcellular response called unfolded protein response (UPR) is induced^4^. UPR comprises three central ER-resident transmembrane proteins: protein kinase R-like endoplasmic reticulum kinase (PERK), inositol-requiring enzyme 1 (IRE1), and activating transcription factor 6 (ATF6), the activation of which ameliorates ER stress by temporarily decreasing 5′-untranslated region (UTR)-dependent translation or up-regulating the transcription of genes in association with ER chaperones^2,3^. On the other hand, when excessive or prolonged ER stress is not relieved intracellularly, UPR removes the abnormal cells from healthy tissue by inducing programmed cell death^5,6^.

Aberrant UPR activation has been implicated in the pathology of various diseases, including diabetes, neurodegeneration, atherosclerosis, and cancer^7^. Thus, the compounds that modulate inevitable ER stress or the pathological activation of UPR may provide the molecular basis for therapy for these diseases^8^. Therefore, we herein investigated small-molecule compounds that suppress UPR, using a library of Myanmar wild plant extracts.

The present results demonstrated that the ethanol extract of the *Periploca calophylla* stem (*P. calophylla* EtOH ex.) inhibited the activation of *X-box binding protein 1 (XBP1)* splicing in a screening assay using HEK293 cells that express the luciferase reporter gene fused with the *XBP1* splicing region. Through activity-guided fractionation and subsequent structural analysis, periplocin^9^ was isolated as an active compound from the methanol extract of the *P. calophylla* stem. Periplocin suppressed IRE1-mediated *XBP1* splicing, activation of the PERK-activating transcription factor 4 (ATF4) pathway, and ATF6 activation involving its fragmentation. In an analysis of the structure–activity relationship, various cardiac glycosides, including periplocin, suppressed *XBP1* splicing. Furthermore, periplocin suppressed the constitutive activation of XBP1 and exerted cytotoxic effects in the human multiple myeloma (MM) cell lines, AMO1 and RPMI8226. The results of the present study indicate the potential of periplocin and other cardiac glycosides as molecular candidates for novel inhibitors of the global UPR pathway.

## Results

### Ethanol extract of P. calophylla stem inhibited the activation of XBP1 splicing

**Figure 1:**
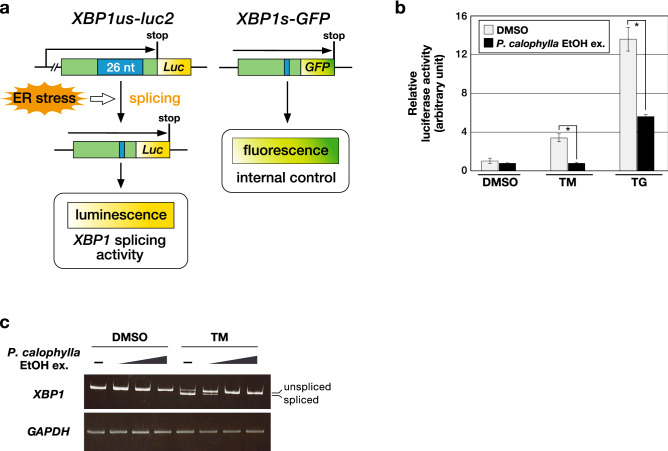
Ethanol extract of the *P. calophylla* stem inhibits activation of the IRE1-XBP1 pathway. (**a**) The scheme of the *XBP1* splicing system (*XBP1us-luc2* and *XBP1s-GFP*) in the reporter construct is shown. (**b**) HEK293 cells, stably expressing both *XBP1us-luc2* and *XBP1s-GFP*, were pretreated with the ethanol extract of the *P. calophylla* stem (*P. calophylla* EtOH ex.) (100 µg/ml) for 1 h, and were then incubated with tunicamycin (TM) (0.5 µg/ml) or thapsigargin (TG) (0.5 µM). DMSO was added as vehicle. After 6 h, luciferase activities and fluorescence intensities in cell lysates were measured. The luciferase activity was normalized by the fluorescence intensity. Values are shown as mean fold activity ± standard deviations (S.D.) (*n* = 3 biological replicates). Statistical analysis was conducted between two groups with or without *P. calophylla* EtOH ex. for each TM or TG treatment assessed by two-tailed Student’s *t*-test, and the significant differences are indicated as *p < 0.05. (**c**) HEK293 cells were pretreated with the *P. calophylla* EtOH ex. in a dose-dependent manner (10, 30, 100 µg/ml) for 1 h, and were then incubated with TM (0.5 µg/ml) for 6 h. The expression of each gene was assessed by RT-PCR. Unspliced or spliced *XBP1* mRNA are indicated. *GAPDH* was used as the loading control. Uncropped images of gels are shown in Supplementary Information, Fig. S7.

To identify the small molecules that suppress UPR activation, we constructed a reporter system that enabled us to illustrate IRE1-mediated *XBP1* splicing. IRE1 is activated by ER stress through autophosphorylation, which results in the splicing out of 26 specific nucleotides from *XBP1* mRNA^10^. We established a cell line stably expressing the *XBP1us-luc2* reporter plasmid in HEK293 cells (Fig. 1a). We used this system to screen a library of 700 Myanmar wild plant extracts to find extracts that inhibit the activation of *XBP1* splicing. The part of results in the screening is shown in Supplementary Fig. S1. In the library, we found that the ethanol extract of *P. calophylla* stem suppressed *XBP1* splicing induced by the ER stressors, tunicamycin (TM) and thapsigargin (TG) (Fig. 1b and Supplementary Fig. S2). The inhibitory effect on the intrinsic splicing of *XBP1* mRNA was also observed by reverse transcription PCR (RT-PCR) (Fig. 1c).

**Figure 2:**
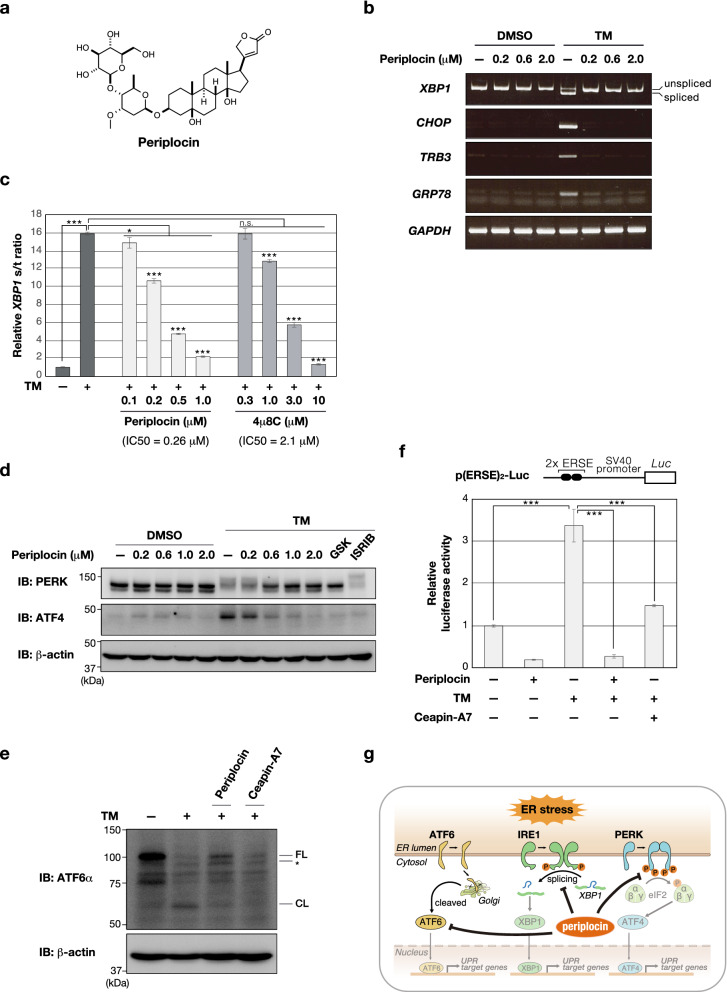
Periplocin suppresses activation of the global UPR pathway. (**a**) Chemical structure of periplocin. (**b**) HEK293 cells were pretreated with the indicated doses of periplocin for 1 h, and were then incubated with TM (0.5 µg/ml) for 6 h. The expression of each gene was assessed by semi-qPCR. Unspliced or spliced *XBP1* mRNA are indicated. *GAPDH* was used as the loading control. (**c**) HEK293 cells were pretreated with the indicated doses of periplocin for 1 h, and were then incubated with TM (0.5 µg/ml) for 6 h. Each expression of spliced *XBP1* mRNA and total *XBP1* mRNA was quantified by real time qPCR. The ratio of spliced *XBP1* mRNA/total *XBP1* mRNA (*XBP1* s/t) are shown as mean fold changes ± S.D. (*n* = 3 technical replicates). IC50s for each of these compounds are shown. Significant differences are indicated as *p < 0.05, ***p < 0.001, assessed by one-way ANOVA with Dunnett’s post-test; n. s., not significant. (**d**) HEK293 cells were pretreated with the indicated doses of periplocin, GSK2656157 (GSK, 1 µM) or ISRIB (0.5 µM) for 1 h, and were then incubated with TM (0.5 µg/ml) for 6 h. Cell lysates were resolved by a 5–20% gradient gel and immunoblotted with the indicated antibodies. β-actin was used as the loading control. One representative from two independent experiments is shown. (**e**) HEK293 cells were pretreated with periplocin (2 µM) or the ATF6α inhibitor Ceapin-A7 (6 µM) for 1 h, and were then incubated with TM (2 µg/ml) for 4 h. The proteasome inhibitor MG132 (10 µM) was added for 1 h prior to cell lysis to avoid degrading ATF6α fragments. Full length of ATF6α (FL), cleaved ATF6α (CL), and non-glycosylated ATF6α (*) are indicated. β-actin was used as the loading control. One representative from two independent experiments is shown. (**f**) A schematic representation of the p(ERSE)_2_-Luc plasmid is shown (top). HEK293 cells were transiently transfected with p(ERSE)_2_-Luc and pCMV/β-gal. After 24 h, cells were pretreated with periplocin (0.2 µM) or Ceapin-A7 (6 µM) for 1 h, and were then incubated with TM (0.5 µg/ml) for 16 h. Luciferase activity in cell lysates was measured and normalized by the β-galactosidase activity. Values are shown as mean fold activity ± S.D. (*n* = 3 biological replicates). Significant differences are indicated as ***p < 0.001, assessed by one-way ANOVA with Dunnett’s post-test. (**g**) A schema for the putative action of periplocin. Uncropped images of gels/blots are shown in Supplementary Information, Fig. S7.

To isolate active compounds from the *P. calophylla* stem, we obtained an extract of the *P. calophylla* stem using methanol, and subsequently performed activity-guided fractionation using the methanol extract. A scheme of the fractionation of the methanol extract is summarized in Supplementary Fig. S3. As a result of fractionation and purification, periplocin (molecular weight: 696)^9^ was characterized as an active compound in the extract of the *P. calophylla* stem (Fig. 2a).

### Periplocin suppresses activation of the whole UPR pathway

We investigated whether periplocin suppresses IRE1-mediated *XBP1* splicing. As shown in Fig. 2b, periplocin suppressed TM-induced *XBP1* mRNA splicing. It is noted that the inhibitory effect on *XBP1* splicing of periplocin was stronger than that of 4µ8C, the inhibitor for IRE1-XBP1 axis^11^ (Fig. 2c). On the other hand, periplocin also inhibited the transcription of *CCAAT/enhancer-binding protein homologous protein (CHOP)*, *tribbles-related protein 3 (TRB3)*, and *glucose-related protein 78 (GRP78)*, the genes of which are induced by the activation of PERK or ATF6 (Fig. 2b). These results prompted us to speculate whether periplocin suppresses not only the IRE1 pathway, but also other UPR pathways including the PERK or ATF6 axis. Therefore, we examined the effects of periplocin on the PERK and ATF6 pathways. PERK is autophosphorylated under ER stress, followed by phosphorylation of eukaryotic initiation factor 2α (eIF2α), increasing selective translation that depend on mRNA containing 5′-UTR, such as ATF4^12^. The results demonstrated that periplocin suppressed the PERK phosphorylation with mobility shift and up-regulation of ATF4 expression (Fig. 2d). These suppressive effects were also observed in cells treated with selective inhibitor GSK2656157, which is well-known to prevent the PERK signaling^13^, indicating periplocin suppresses the PERK pathway. We also examined the effect of an integrated stress response inhibitor (ISRIB), which was characterized that blunts the integrated stress response (ISR) by antagonizing the inhibitory effect of phosphorylated eIF2 on eIF2B^14^. It should be noted that pretreatment of ISRIB suppressed ATF4 induction but not PERK phosphorylation under ER stress (Fig. 2d). Furthermore, to investigate whether periplocin prevents ATF6 pathway, we detected ATF6α protein fragmentation and ATF6-transcriptional activity using a reporter plasmid including the ER stress element (ERSE)^15^. As shown in Fig. 2e, the specific ATF6α inhibitor Ceapin-A7^16^ decreased the ATF6α cleaved fragment (shown as CL, 50–75 kDa). Similarly, pretreatment with periplocin reduced the cleaved ATF6α bands (Fig. 2e). In addition, periplocin abrogated the TM-induced response of ERSE as well as Ceapin-A7 (Fig. 2f). These results indicate that periplocin suppressed both the PERK and ATF6 pathways, suggesting that it suppresses the activation of not only the IRE1 pathway, but also both the PERK and ATF6 pathways (Fig. 2g).

### Cardiac glycosides and their aglycones suppress activation of the IRE1-XBP1 pathway

**Figure 3:**
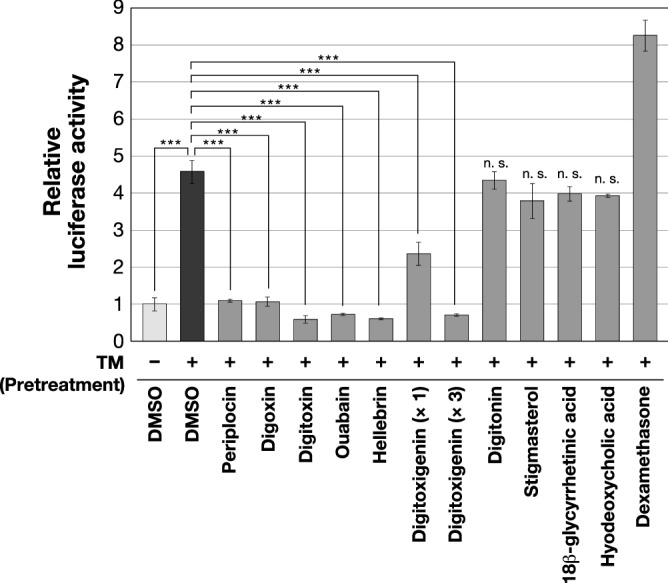
Various cardiac glycosides exert suppressive effects on UPR. HEK293 cells, stably expressing both *XBP1us-luc2* and *XBP1s-GFP*, were pretreated with cardiac glycosides (0.2 µM): periplocin, digoxin, digitoxin, ouabain, and hellebrin, or an aglycone (× 1: 0.2 µM, × 3: 0.6 µM): digitoxigenin, or the other compounds that have a steroidal back bone, but do not exhibit cardiotonic activity (0.2 µM): digitonin, stigmasterol, 18*β*-glycyrrhetinic acid, hyodeoxycholic acid, and dexamethasone, for 1 h and were then incubated with TM (0.5 µg/ml). DMSO was added as vehicle. After 6 h, luciferase activities and fluorescence intensities in cell lysates were measured. Luciferase activity was normalized by fluorescence intensity. Values shown are mean fold activity ± S.D. (*n* = 3 biological replicates). Significant differences are indicated as ***p < 0.001, assessed by one-way ANOVA with Dunnett’s post-test; n. s., not significant.

Periplocin, a member of cardenolides, was initially isolated from *Cortex periplocae* root bark^17,18^. Periplocin, like digoxin, digitoxin, and ouabain, belongs to a group with a chemical structure containing a steroid skeleton and carbohydrate moiety^19^. These compounds in the group are categorized as cardiac glycosides. Therefore, we assumed that other cardiac glycosides may also inhibit UPR activation. To elucidate this assumption, we evaluated the inhibitory activities on *XBP1* splicing of cardiac glycosides or compounds containing steroid skeletons (these structures are shown in Supplementary Fig. S4). The results of the luciferase assay in *XBP1us-luc2* stably expressing cells showed that digoxin, digitoxin, ouabain, and hellebrin suppressed *XBP1* splicing at concentrations similar to periplocin. This suppressive effect was also observed with digitoxigenin, which is an aglycone of the digitoxin; however, its effects appeared to be weaker than that of the glycosides. On the other hand, other compounds with a steroid skeleton but not included in cardiotonic steroids, such as digitonin, stigmasterol, 18*β*-glycyrrhetinic acid, hyodeoxycholic acid, and dexamethasone, did not suppress *XBP1* splicing (Fig. 3). These results indicate that cardiac glycosides and their aglycones exhibit inhibitory activity against IRE1-mediated *XBP1* splicing.

### Inhibitory effects of periplocin on constitutive activation of the IRE1-XBP1 pathway in multiple myeloma cells and its cytotoxic effect on cells

**Figure 4:**
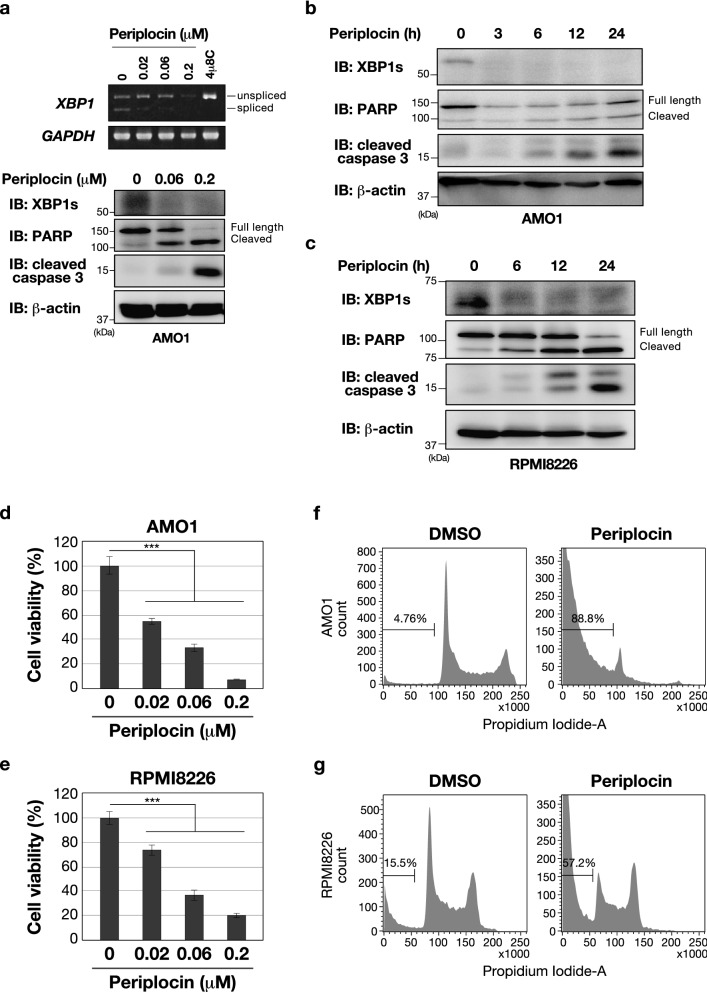
Periplocin inhibits the constitutive activation of the IRE1-XBP1 pathway in multiple myeloma cells and exerts a cytotoxic effect on them. (**a**) For upper panel: AMO1 cells were incubated with the indicated doses of periplocin or 4 µ8C (10 µM) for 24 h. The expression of each gene was assessed by RT-PCR. Unspliced or spliced *XBP1* mRNA are indicated. *GAPDH* was used as the loading control. For lower panel: AMO1 cells were incubated with the indicated doses of periplocin for 24 h. Cell lysates were immunoblotted with the indicated antibodies. β-actin was used as the loading control. In the immunoblotting one representative from two independent experiments is shown. (**b, c**) AMO1 (**b**) and RPMI8226 (**c**) cells were incubated with periplocin (0.2 µM) for the indicated times. Cell lysates were immunoblotted with the indicated antibodies. β-actin was used as the loading control. One representative from two independent experiments is shown. (**d, e**) AMO1 (**d**) and RPMI8226 (**e**) cells were incubated with the indicated doses of periplocin for 24 h. Cell viability was measured by the WST-8 cell proliferation assay. Results are shown as mean ± S.D. (*n* = 3 biological replicates). Significant differences are indicated as ***p < 0.001, assessed by one-way ANOVA with Dunnett’s post-test. (**f**,**g**) AMO1 (**f**) and RPMI8226 (**g**) cells were incubated with periplocin (0.2 µM) or DMSO. After 24 h, cells were stained with propidium iodide for a flow cytometric analysis of DNA content. The ratio of cells with DNA degradation is shown as a sub-G1 fraction and percentages are indicated in the histogram. Uncropped images of gels/blots are shown in Supplementary Information, Fig. S7.

The IRE1-XBP1 pathway has been reported to be persistently activated and contribute to pathogenesis in some cancerous contexts. For example, survival or proliferation of multiple myeloma (MM) cells has been suggested to depend on the IRE1 axis. Indeed, the inhibition of *XBP1* mRNA splicing was shown to induce apoptosis in MM cells^20,21^. The present results clearly demonstrated that periplocin inhibited the splicing of *XBP1* mRNA caused by treatment with ER stressors (Figs. 1, 2 and 3). Moreover, periplocin has been reported to exhibit antitumor activity against several cancer cells^19^. These results led us to assume that periplocin showed suppressive effects on MM cell survival. To confirm the hypothesis, we investigated whether periplocin induced apoptosis in several MM cell line. In myeloma AMO1 and RPMI8226 cells, constitutive XBP1s expression was detected even under the absence of TM (Supplementary Fig. S5a). We also confirmed the position of XBP1s protein bands by HEK293 cells incubated with TM in the absence or presence of 4µ8C (Supplementary Fig. S5b). Notably, the constitutive splicing of *XBP1* mRNA and expression of XBP1s protein in AMO1 myeloma cells were inhibited in a dose-dependent manner following an exposure to periplocin (Fig. 4a). Periplocin also suppressed XBP1s protein levels with its time-dependent treatment in both AMO1 and RPMI8226 myeloma cells (Fig. 4b,c). Furthermore, dose- or time-dependent exposure of periplocin to these cells increased the level of truncated PARP and caspase 3, which are apoptosis markers (Fig. 4a–c). The viability of these cells was significantly reduced by the dose-dependent treatment with periplocin (Fig. 4d,e). Strikingly, the periplocin treatment increased the sub-G1 fraction including DNA-fragment of both AMO1 and RPMI8226 cells (Fig. 4f,g). These results suggest that periplocin has the potential to suppress *XBP1* splicing and reduce MM cell viability. In contrast, as shown in Supplementary Fig. S6, periplocin had little effect on the viability of normal human fibroblasts, TIG-1 cells. The constitutive splicing of *XBP1* mRNA was not observed in TIG-1 cells (Supplementary Fig. S6). Collectively, the present results indicate that periplocin inhibits the ER stress-induced and constitutive activation of the IRE1-XBP1 pathway in MM cells. In addition, periplocin induced apoptosis in MM cells, but only had a negligible effect on normal cells.

## Discussion

Recent findings indicated that excessive or chronical ER stress followed by UPR activation contributes to the pathogenesis of various diseases^7^. Thus, small molecules modulating UPR may help elucidate the relationship between UPR and diseases. Furthermore, approaches to control UPR activation are expected to contribute to the development of novel therapeutic interventions for UPR-related disorders, such as inflammatory diseases and cancer^8^. Therefore, we herein attempted to identify novel active compounds that suppress UPR by monitoring the inhibitory effects on the IRE1-XBP1 pathway. We have succeeded in isolating periplocin from the methanol extract of *P. calophylla* as a potent inhibitor of the IRE1-XBP1 pathway.

We found that periplocin suppressed the activation of all three UPR branches in HEK293 cells (Fig. 2). For this reason, periplocin appears to affect the upstream pathway of UPR. In addition, the ethanol extract of *P. calophylla* or its active ingredient, periplocin, individually suppressed IRE1-mediated *XBP1* splicing induced by different types of ER stressors TM or TG, respectively (Fig. 1, 2). These results suggest that periplocin affects the factor(s) that modulates global UPR signaling, such as ER stress. Previous studies have reported that several compounds suppress UPR signaling^11,16,22^. 4-phenyl butyrate (4-PBA) or tauroursodeoxycholic acid (TUDCA), which is ER stress inhibitor, attenuates ER stress resulting in suppression of UPR activation^23–25^. Of note, unlike these compounds, periplocin is classically known to suppresses Na/K-ATPase activity as a cardiac glycoside^19,26^. However, pharmacological effect of suppressing Na/K-ATPase on UPR activation has been poorly elucidated. Interestingly, recent reports have shown that cardiac glycosides interact with Na/K-ATPase to modulate cellular signaling pathway, such as nuclear factor (NF)-κB axis or mitogen-activated protein kinase (MAPK) signaling^26^. Therefore, the suppressive effect of periplocin on UPR may be due to the regulation of Na/K-ATPase. To support this hypothesis, as shown in Fig. 3, only the cardiac glycosides periplocin, digoxin, digitoxin, ouabain, and hellebrin and an aglycone (digitoxigenin) suppressed IRE1-XBP1 activity. The suppressive effect of the aglycone on the IRE1-XBP1 axis was weaker than those of glycosides. Previous studies showed that the glycoside form of cardiac glycosides exhibited a stronger binding affinity to Na/K-ATPase than that of aglycones, which is more likely to influence inhibitory efficacy^26,27^. These findings indicate that the structural properties in cardiac glycosides required to depress Na/K-ATPase are key factors in inhibiting UPR. Although we did not clarify the protein(s) that periplocin targeted in UPR, the future identification of binding protein(s) for periplocin will provide insights into the novel molecular factor(s) regulating UPR.

In the UPR pathway, the IRE1-XBP1 axis has been shown to be associated with cancer progression and malignancy^28,29^. In drug-resistant malignant tumors, *XBP1* mRNA is continuously spliced, and XBP1 modulates a number of genes associated with angiogenesis, cell proliferation, and metastasis^30,31^. Moreover, the IRE1 pathway has been implicated in MM. Munshi et al*.* showed that *XBP1* mRNA was strongly expressed in patient-derived MM cells^32^. Furthermore, transgenic mice overexpressing XBP1 exhibited pathological conditions similar to MM^33^. These findings indicate that the IRE1-XBP1 axis plays an essential role in the pathological mechanisms of several cancers including MM, and, hence, inhibitors for the IRE1-XBP1 pathway are currently being developed^34^. For example, toyocamycin and MKC-3946, which are small molecules that prevent IRE1 from splicing *XBP1* mRNA, have the potential to depress cell proliferation and/or induce apoptosis in MM cells^20,21^. Similarly, our results using MM cells showed that periplocin inhibited constitutive *XBP1* splicing, induced apoptotic cell death, and suppressed cell proliferation (Fig. 4). Previous studies on XBP1 silencing have reported marked reductions in the proliferation of MM cells^21^. These findings suggest that anticancer effects in MM cells exposed to periplocin were derived from the inhibition of the IRE1-XBP1 pathway.

In conclusion, periplocin suppressed the global UPR pathway by acting on unidentified factors. UPR signaling appears to be directly or indirectly associated with Na/K-ATPase, based on the structural properties or active moieties of periplocin and other cardiac glycosides. Periplocin also suppressed the survival of MM cells in which the IRE1-XBP1 pathway was constitutively activated. In addition to these suppressive effects, previous studies have indicated that periplocin has a variety of pharmacological properties, such as cardiotonic effects and anticarcinogenic and proapoptotic activities^17,18^. Several epidemiological studies have demonstrated that a treatment with cardiac glycosides prevented cancer recurrence and prolonged survival of cancer patients^35^. The suppression of the IRE1-XBP1 pathway by cardiac glycosides may effectively improve the prognosis of cancer patients, and this will be clarified in future studies. Further investigations will provide novel insights into the modes of action of periplocin by identifying its targeted protein(s), which may reveal mechanisms contributing to the physiological or pathological regulation of UPR.

## Methods

### Cell culture, plasmids, and establishment of a stable cell line

HEK293 and TIG1 cells were maintained in Dulbecco’s modified Eagle’s medium (DMEM) (Nacalai Tesque, Kyoto, Japan) supplemented with 10% fetal bovine serum (FBS) (Sigma, St. Louis, MO, USA), 100 U/ml of penicillin G, and 100 µg/ml of streptomycin^36,37^. AMO1 and RPMI8226 cells were cultured in Roswell Park Memorial Institute (RPMI) 1640 medium (Sigma) containing 10% FBS and penicillin/streptomycin. Cells were grown in a 5% CO_2_ atmosphere at 37 °C.

*XBP1us*-*luc2* was constructed by ligating pcDNA3.1-Hygro (Invitrogen, Carlsbad, CA, USA) with an unspliced form of a Flag-*XBP1*ΔDBD fragment^38^, followed by the *luc2* reporter gene derived from pGL4.10 (Promega, Madison, WI, USA). *XBP1s*-*GFP* was constructed by ligating pEGFP-N1 (TaKaRa Bio Inc., Shiga, Japan) with a spliced form of the Flag-*XBP1*ΔDBD fragment. p(ERSE)_2_-Luc was obtained from two tandem repeats of ERSE, which were amplified by PCR and ligated with the pGL3-Promoter Vector (Promega)^39^. pCMV/ β-gal was used as a plasmid expressing β-galactosidase (β-gal).

HEK293 cells were transfected with *XBP1us*-*luc2* using polyethylenimines (Polysciences, Warrington, PA, USA). After 48 h, cells were selected with 100 µg/ml hygromycin. When colonies derived from a single cell were obtained, we subsequently transfected cells with *XBP1s-GFP*. Clonal cells expressing both *XBP1us-luc2* and *XBP1s-GFP* were used in experiments.

### RNA extraction, semi-quantitative PCR (semi-qPCR), and real time qPCR (qPCR)

Total RNA was extracted from cells as described previously^40^. First-strand cDNA was synthesized with the Prime Script first-strand cDNA Synthesis Kit (TaKaRa Bio Inc.)^40^. Semi-qPCR was performed as described previously^41^, using Gene Amp PCR System 2700 (Applied Biosystems, Foster City, CA, USA). Primers used for PCR were as follows: human *CHOP*, 5′ -GCGTCTAGAATGGCAGCTGAGTCATTGCC-3′ (forward) and 5′ -GCGTCTAGATCATGCTTGGTGCAGATTC-3′ (reverse); human *TRB3*, 5′ -TGCCCTACAGGCACTGAGTA-3′ (forward) and 5′ -GTCCGAGTGAAAAAGGCGTA-3′ (reverse); human *glyceraldehyde-3-phosphate dehydrogenase* (*GAPDH)*, 5′ -TGAAGGTCGGAGTCAAAGGATTTGGT-3′ (forward) and 5′ -CATGTGGGCCATGAGGTCCACCAC-3′ (reverse); human *XBP1* (for Fig. 1c, 2b, Supplementary Fig. S6), 5′ -GGAGTTAAGACAGCGCTTGG-3′ (forward) and 5′ -ACTGGGTCCAAGTTGTCCAG-3′ (reverse); human *XBP1* (for Fig. 4a), 5′ -CGGAAGCCAAGGGGAATGAAG -3′ (forward) and 5′ -GGATATCAGACTCTGAATC-3′ (reverse); human *GRP78*, 5′ -TGAAGAGCTCAACATGGATCTGTT-3′ (forward) and 5′ -CTACAGCTTCATCTGGGTTTATGC-3′ (reverse). A semiquantitative analysis for PCR products was performed using 8% polyacrylamide gel (for *XBP1*) or 1.5–2% agarose gel (for the others) electrophoresis. All the original data acquisition of gels is included in a Supplementary Fig. S7. qPCR was performed as described previously^42^. The specificities of the detected signals were confirmed by a dissociation curve, which consisted of a single peak. The primers used for qPCR were as follows: human *XBP1*s (spliced), 5′-CTGAGTCCGCAGCAGGTG-3′ (forward) and 5′-TCCAAGTTGTCCAGAATGCC-3′ (reverse); human *XBP1*t (total), 5′-GGCATCCTGGCTTGCCTCCA-3′ (forward) and 5′-GCCCCCTCAGCAGGTGTTCC-3′ (reverse). The rate of spliced *XBP1* mRNA/total *XBP1* mRNA were calculated to show in Fig. 2c.

### Immunochemical methods and antibodies

Immunoblotting was conducted as previously described^43^. In Fig. 2c, a gradient gel (SuperSep™ Ace, 5–20%; FUJIFILM Wako) was used for separating protein samples. The following commercially available antibodies were used: anti-PERK (#5683; Cell Signaling Technology, Beverly, MA, USA), anti-ATF4 (#11815; Cell Signaling Technology), anti-ATF6α (73-500; BioAcademia), anti-XBP1s (#12782; Cell Signaling Technology), anti-PARP (#9542; Cell Signaling Technology), anti-Cleaved Caspase-3 (Asp175) (#9664; Cell Signaling Technology) and anti-β-actin (A5441; Sigma). All the original data acquisition of western blots is included in a Supplementary Fig. S7.

### Reporter assay

HEK293 cells were transfected with the luciferase-expressing reporter, β-galactosidase, and empty vector. After 24 h, cells were treated for the indicated periods with ER stressors. The luciferase assay was performed as previously described^40^. Values of luciferase activity were normalized to β-gal activity or the fluorescence intensity of GFP.

### Cell viability assay and cell death assay

A cell proliferation assay was performed using water-soluble tetrazolium (WST)-8 (Dojindo, Kumamoto, Japan), as previously described^44^. AMO1 and RPMI8226 cells were plated at a concentration of 1 × 10^4^ cells per well on a 96-well plate. Cells were then treated with periplocin for 24 h. The WST-8 reagent was added and cells were incubated at 37 °C for 3 h in a 5% CO_2_ atmosphere. Absorbance at 450 nm of the medium was measured. The percentage of cell proliferation was normalized to vehicle-treated cells. Regarding the quantification of cell death, TIG1 cells were trypsinized and stained with trypan blue (Invitrogen) followed by counting with a hemocytometer. Cells stained blue were regarded as dead cells.

### Sub-G1 fraction analysis

Cells were stained with propidium iodide using the Cycletest™ Plus DNA Kit (BD Biosciences, Franklin Lakes, NJ, USA) according to the manufacturer’s instructions. Changes in the DNA content in cells were detected using the FACSVerse™ flow cytometer (BD Biosciences). The percentage of cells in the sub-G1 fraction was analyzed by FACSuite™ software (BD Biosciences).

### Myanmar natural plant extract library and procedure of screening

The plant materials used to construct the 700 types of extract library, including the *P. calophylla* stem, were collected in Myanmar under the Memorandum of Understanding between the Kochi Prefectural Makino Botanical Garden (MBK), Japan and the Forestry Department (FD), the Ministry of Natural Resources and Environmental Conservation, Myanmar, in accordance with the Convention on Biological Diversity (CBD) and the Nagoya Protocol on access to genetic resources and benefit sharing. FD is the National Focal Point of Myanmar on CBD. Plant inventory in Myanmar has been performed under the permission from the Director General of FD. Voucher specimens of plant materials were deposited in the herbarium of MBK. Plant samples were equipped as an ethanol extract to the laboratory. Before use in screening, they were dissolved in DMSO to 100 mg/ml. All extracts (100 µg/ml) were applied to cells expressing both *XBP1us-luc2* and *XBP1s-GFP* for 1 h prior to incubation with tunicamycin (0.5 µg/ml) for 6 h, and then the cell lysates were obtained. The *XBP1* splicing activity was evaluated by the value of luciferase activity normalized by fluorescence intensity in the cell lysates. The extracts which decreased more than 50% of tunicamycin-induced *XBP1* splicing were determined as positive candidates in this screening.

### Plant material

*P. calophylla* (Wight) Falc. was collected in the CHIN State in Myanmar. Botanical identification was performed by Dr. Kazumi Fujikawa, MBK.

### Spectroscopic experimental procedures

Optical rotation was recorded on a JASCO P-2100 polarimeter. NMR spectra were recorded on an Agilent Varian VNS500 spectrometer. Chemical shifts (ppm) were referenced to residual solvent peaks (δ_H_ 3.31 and δ_C_ 49.0 for CD_3_OD). Positive-mode ESITOFMS was obtained on a JEOL JMS-T100LP AccuTOF LC-plus 4G spectrometer using a sample dissolved in MeOH.

### Extraction and isolation of periplocin from P. calophylla stem

The dried stem of *P. calophylla* (163 g) was extracted with methanol at room temperature and evaporated *in vacuo*. The concentrated methanol extract (5.17 g) was successively partitioned among ethyl acetate, 1-butanol, and H_2_O to give active residues of the ethyl acetate and 1-butanol fractions, and inactive residues of the H_2_O fractions. The 1-butanol-soluble fraction was separated over a silica gel column using a stepwise gradient of increasing polarity from 100 to 0% chloroform in methanol into 11 sub-fractions. Two 1-butanol fractions (fraction 4, 5) were separated over an ODS-modified silica gel column using a stepwise gradient of decreasing polarity from 20 to 100% methanol aqueous solution into 10 sub-fractions. One of the 10 sub-fractions (fraction 7: 25.7 mg) was detected to have the potential to inhibit *XBP1* splicing activity using a reporter assay. 1D and 2D NMR and ESI–MS analyses clarified that the active compound responsible for suppressing *XBP1* splicing was periplocin. Periplocin: [α]_D_^27^ + 11 (*c* 0.5, EtOH); ^1^H-NMR (CD_3_OD, 500 MHz) *δ* 5.90 (1H, brs, H-22), 5.03 (1H, dd 18.5, 2.0 Hz, H-21a), 4.91 (1H, dd 18.5, 2.0 Hz, H-21b), 4.86 (1H, m, H-1′), 4.34 (1H, d 7.5 Hz, H-1″), 4.15 (1H, m, H-3), 3.94 (1H, ddd 3.0, 3.0, 3.0 Hz), 3.90 (1H, m, H-6″a), 3.88 (1H, m, H-5′), 3.64 (1H, dd 12.0, 6.0 Hz, 6″b), 3.46 (3H, s, H-7′), 3.35 (1H, m, H-4′), 3.35 (1H, m, H-3″), 3.27 (1H, m, H-5″), 3.24 (1H, m, H-4″), 3.20 (1H, dd 9.5, 7.5 Hz, H-2″), 2.84 (1H, m, H-17), 2.18 (1H, m, H-4a), 2.16 (1H, m, H-16a), 2.14 (1H, m, H-15a), 2.10 (1H, brd 13.5 Hz, H-2′), 1.93 (1H, m, H-7a), 1.88 (1H, m, 16b), 1.78 (1H, m, H-2a), 1.72 (1H, m, H-6a), 1.72 (1H, m, H-15b), 1.71 (1H, m, H-1a), 1.66 (1H, m, H-2b), 1.65 (1H, m, H-8), 1.63 (1H, m, H-2′b), 1.61 (1H, m, H-9), 1.56 (1H, m, H-4b), 1.50 (2H, H-12), 1.47 (1H, m, H-11a), 1.39 (1H, m, H-1b), 1.35 (1H, m,. H-6b), 1.32 (1H, m, H-11b), 1.29 (3H, d 6.5 Hz, H-6′), 1.21 (1H, dddd 11.5, 11.5, 11.5, 4.0 Hz), 0.93 (3H, s, H-19), 0.88 (3H, s, H-18); ^13^C-NMR (CD_3_OD, 125 MHz) *δ* 178.3 (C-20), 177.2 (C-23), 117.8 (C-22), 106.2 (C-1″), 98.1 (C-1′), 86.3 (C-14), 83.7 (C-4′), 78.6 (C-3′), 78.0 (C-5″), 77.9 (C-3″), 77.2 (C-3), 75.8 (C-5), 75.3 (C-21), 75.3 (C-2″), 71.8 (C-4″), 70.3 (C-5′), 63.0 (C-6″), 58.6 (C-7′), 51.9 (C-17), 50.9 (C-13), 41.8 (C-10), 41.7 (C-8), 40.9 (C-12), 40.2 (C-9), 36.6 (C-2′), 35.8 (C-4), 35.5 (C-6), 33.3 (C-15), 28.0 (C-16), 26.8 (C-2), 26.6 (C-1), 24.8 (C-7), 22.7 (C-11), 18.6 (C-6′), 17.2 (C-19), 16.3 (C-18); ESITOFMS *m/z* 719 (M + Na)^+^; HRESITOFMS m/z 719.3622 [M + Na]^+^ (calcd for C_36_H_56_O_13_Na, 719.3619).

### Chemicals

Periplocin (CAS: 13137-64-9) was commercially obtained from ChemFaces (Wuhan, China). Digoxin and digitoxin were purchased from Tokyo Kasei Kogyo (Tokyo, Japan). GSK2656157, ISRIB, digitoxigenin, and hellebrin were purchased from Cayman Chemical (Ann Arbor, MI, USA). The other chemicals were purchased from Sigma.

### Statistical analysis

The significance of differences between two groups was assessed by the two-tailed Student’s *t*-test. An analysis of significance for multiple groups was performed by a one-way ANOVA with the post hoc Dunnett’s test.

## Supplementary information

Supplementary Information.
