# Supplementary material for: Periplocin and cardiac glycosides suppress the unfolded protein response
Source: Sci Rep. 2021 May 4;11:9528. doi: 10.1038/s41598-021-89074-x (PMC8097017; doi:10.1038/s41598-021-89074-x)
Supplement: Supplementary file 1 — Supplementary Information. [file 41598_2021_89074_MOESM1_ESM.pdf]

**Periplocin and cardiac glycosides suppress the unfolded protein response**

Muneshige Tokugawa[1], Yasumichi Inoue[1,2], Kan'ichiro Ishiuchi[3], Chisane Kujirai[1], Michiyo Matsuno[4], Masaki Ri[5], Yuka Itoh[1], Chiharu Miyajima[1,2], Daisuke Morishita[1,6], Nobumichi Ohoka[7], Shinsuke Iida[5], Hajime Mizukami[4], Toshiaki Makino[3], Hidetoshi Hayashi[1,2]

[1]Department of Cell Signaling, Graduate School of Pharmaceutical Sciences, Nagoya City University, Nagoya 467-8603, Japan

[2]Department of Innovative Therapeutic Sciences, Cooperative Major in Nanopharmaceutical Sciences, Graduate School of Pharmaceutical Sciences, Nagoya City University, Nagoya 467-8603, Japan

[3]Department of Pharmacognosy, Graduate School of Pharmaceutical Sciences, Nagoya City University, Nagoya 467-8603, Japan

[4]The Kochi Prefectural Makino Botanical Garden, Kochi 781-8125, Japan

[5]Department of Hematology and Oncology, Graduate School of Medical Sciences, Nagoya City University, Nagoya 467-8601, Japan

[6]Chordia Therapeutics Inc., Kanagawa 251-0012, Japan

[7]Division of Molecular Target and Gene Therapy Products, National Institute of Health Sciences, Kawasaki 210-9501, Japan

**a**

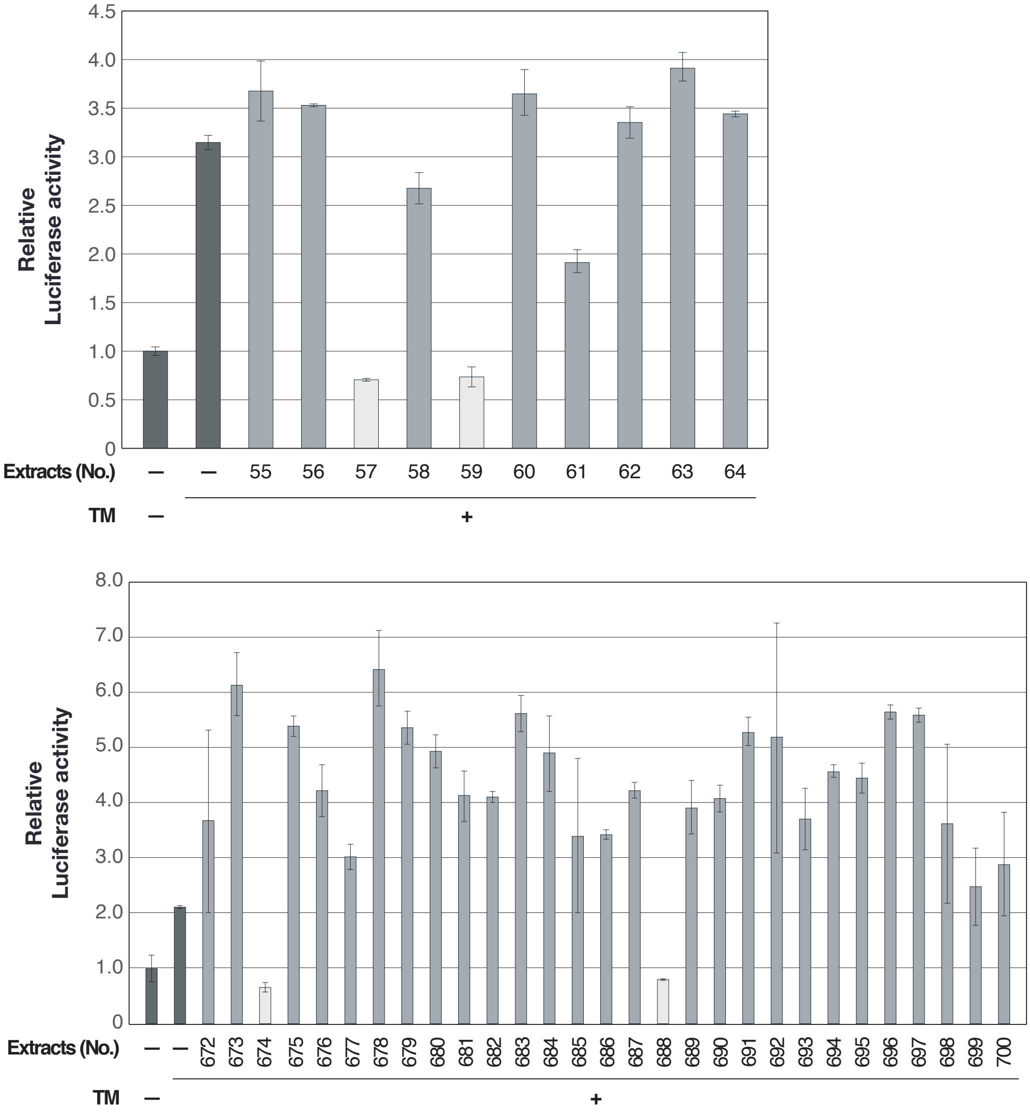


## Table 1

***Positive candidates in Myanmar plants extracts screening***

| Sample No. | Family | Species | Part |
|---|---|---|---|
| 57 | Asclepiadaceae | *Periploca calophylla* | Stem |
| 59 | Aristolochiaceae | *Aristolochia wardiana* | Root |
| 674 | Taxaceae | *Cephalotaxus mannii* | Trunk (stem) |
| 688 | Rubiaceae | *Rubia garrettii* | Root |

**Supplementary Figure S1** The part of results and positive candidates in screening the Myanmar wild plants extracts to inhibit *XBP1* splicing activity.
(**a**) HEK293 cells, stably expressing both *XBP1us-luc2* and *XBP1s-GFP*, were pretreated with the ethanol extracts of Myanmar wild plants (100 μg/ml) for 1h prior to incubation with tunicamycin (TM) (0.5 μg/ml) for 6 h. After the incubation, luciferase activities and fluorescence intensities in cell lysates were measured. The luciferase activity was normalized by the fluorescence intensity. Values are shown as mean fold activity ± standard deviations (*n* = 3 biological replicates). (**Table 1**) The positive candidates of Myanmar plants extracts are shown. The sample No. 57 was determined to purify and explore the active ingredient in this study. The detailed procedure for screening is also explained in Methods.

**Supplementary Figure S1**

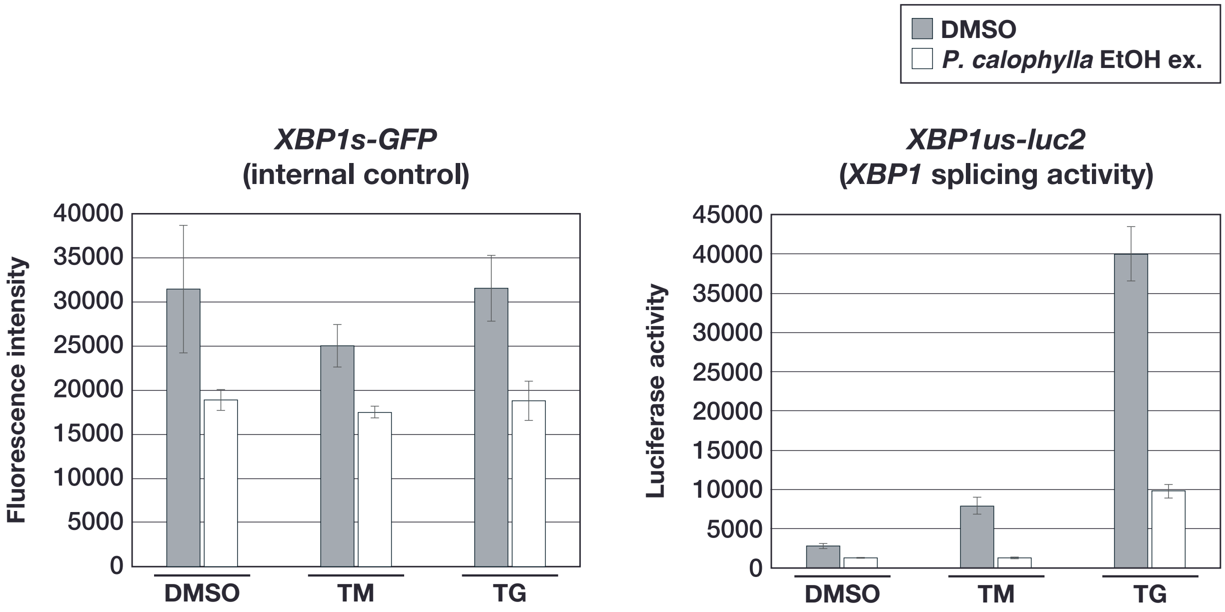


**Supplementary Figure S2** The influence on luciferase activity and fluorescence intensity by treatment with *Periploca calophylla* stem EtOH extracts. These data are corresponded to Figure 1b.

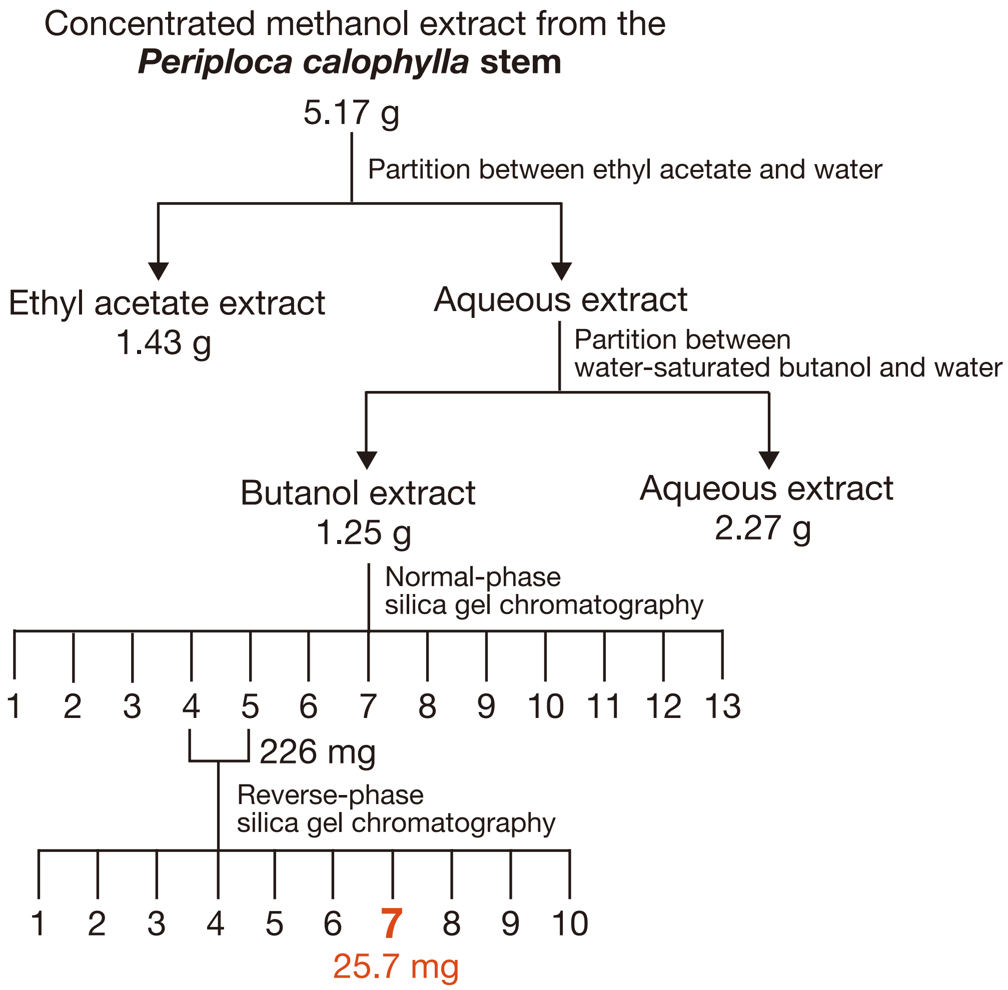


**Supplementary Figure S3** Activity-guided fractionation of the methanol extract of *P. calophylla* stem and isolation of active principles.

**Supplementary Figure S3**

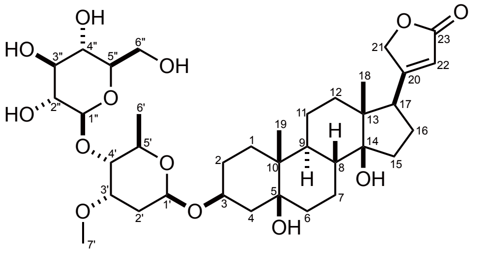

(1) Periplocin

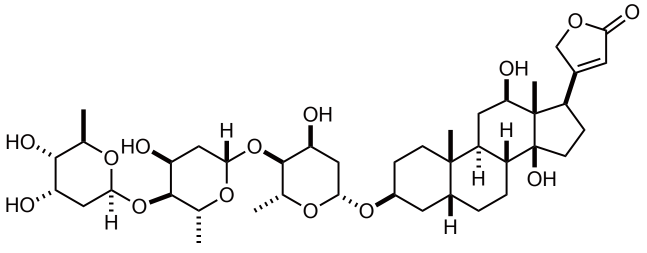

(2) Digoxin

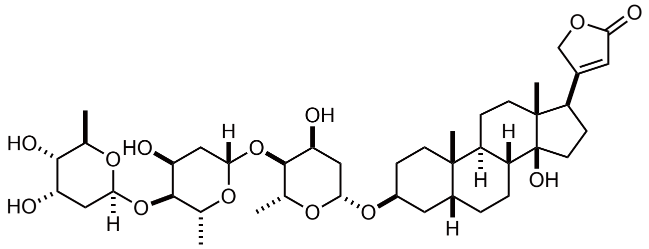

(3) Digitoxin

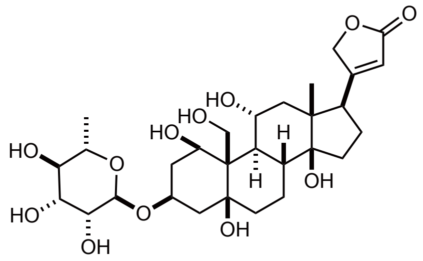

(4) Ouabain

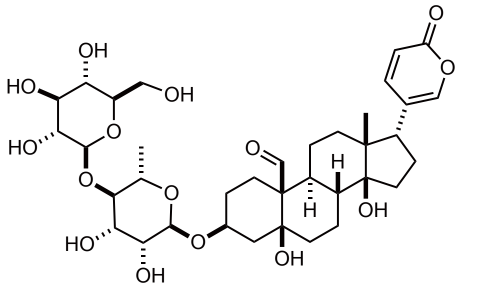

(5) Hellebrin

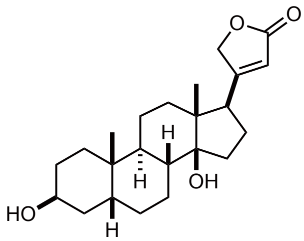

(6) Digitoxigenin

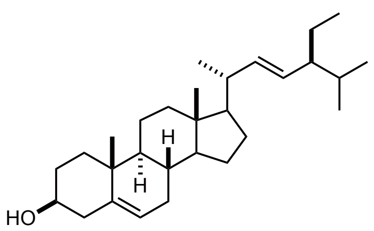

(7) Stigmasterol

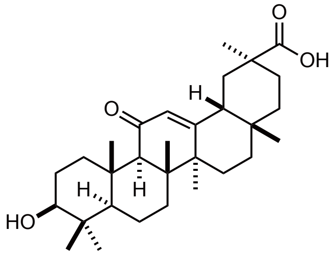

(8) 18β-Glycyrrhetinic acid

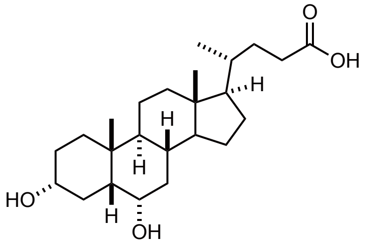

(9) Hyodeoxycholic acid

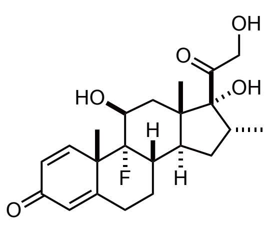

(10) Dexamethasone

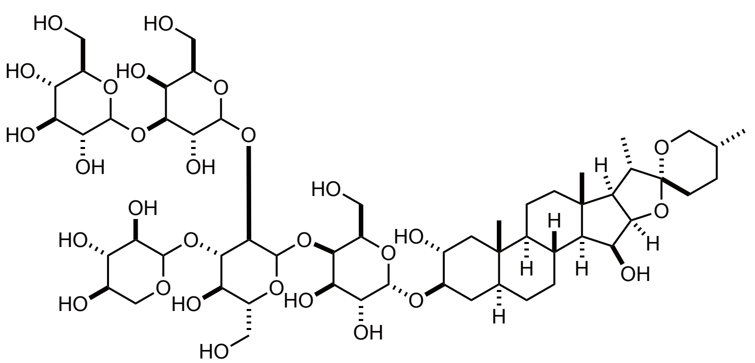

(11) Digitonin

**Supplementary Figure S4** The structure of all cardiac glycosides or its analogs used in Fig. 3

**Supplementary Figure S4**

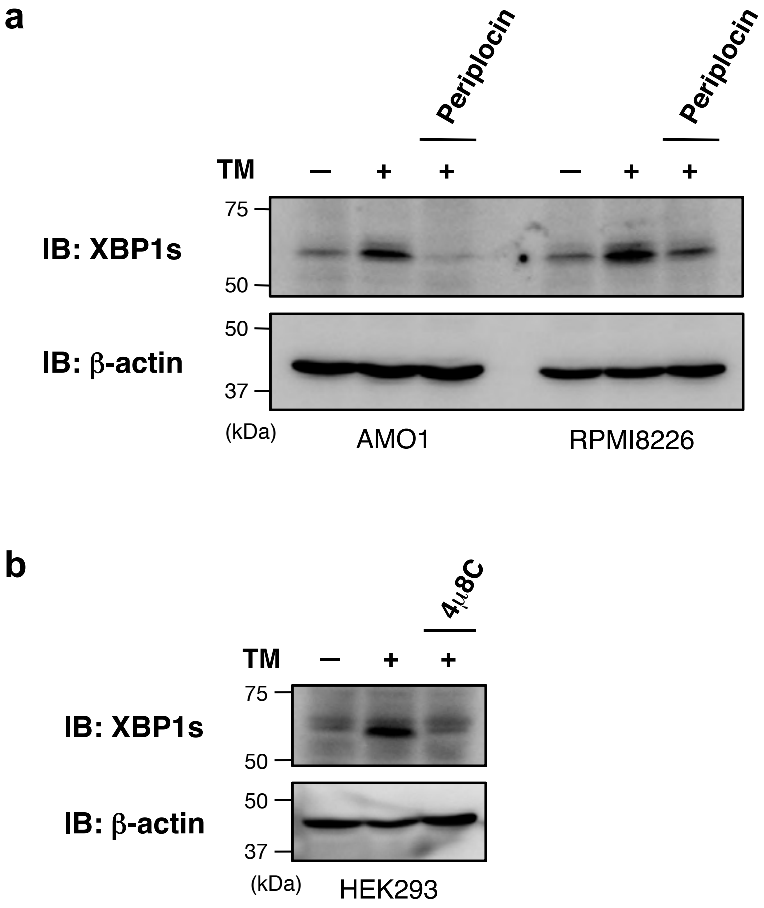


**Supplementary Figure S5** Identification of intrinsic XBP1s expression in multiple myeloma cells.
(**a**) Multiple myeloma AMO1 and RPMI8226 cells were incubated with or without periplocin (0.2 μM) for 1 h followed by treatment with tunicamycin (TM, 0.5 μg/ml) for 4 h. Cell lysates were immunoblotted with the indicated antibodies. β-actin was used as the loading control. (**b**) HEK293 cells were incubated with or without 4μ8C (10 μM) for 1h followed by treatment with TM (0.5 μg/ml) for 6 h. Cell lysates were immunoblotted with the indicated antibodies. β-actin was used as the loading control.

**Supplementary Figure S5**

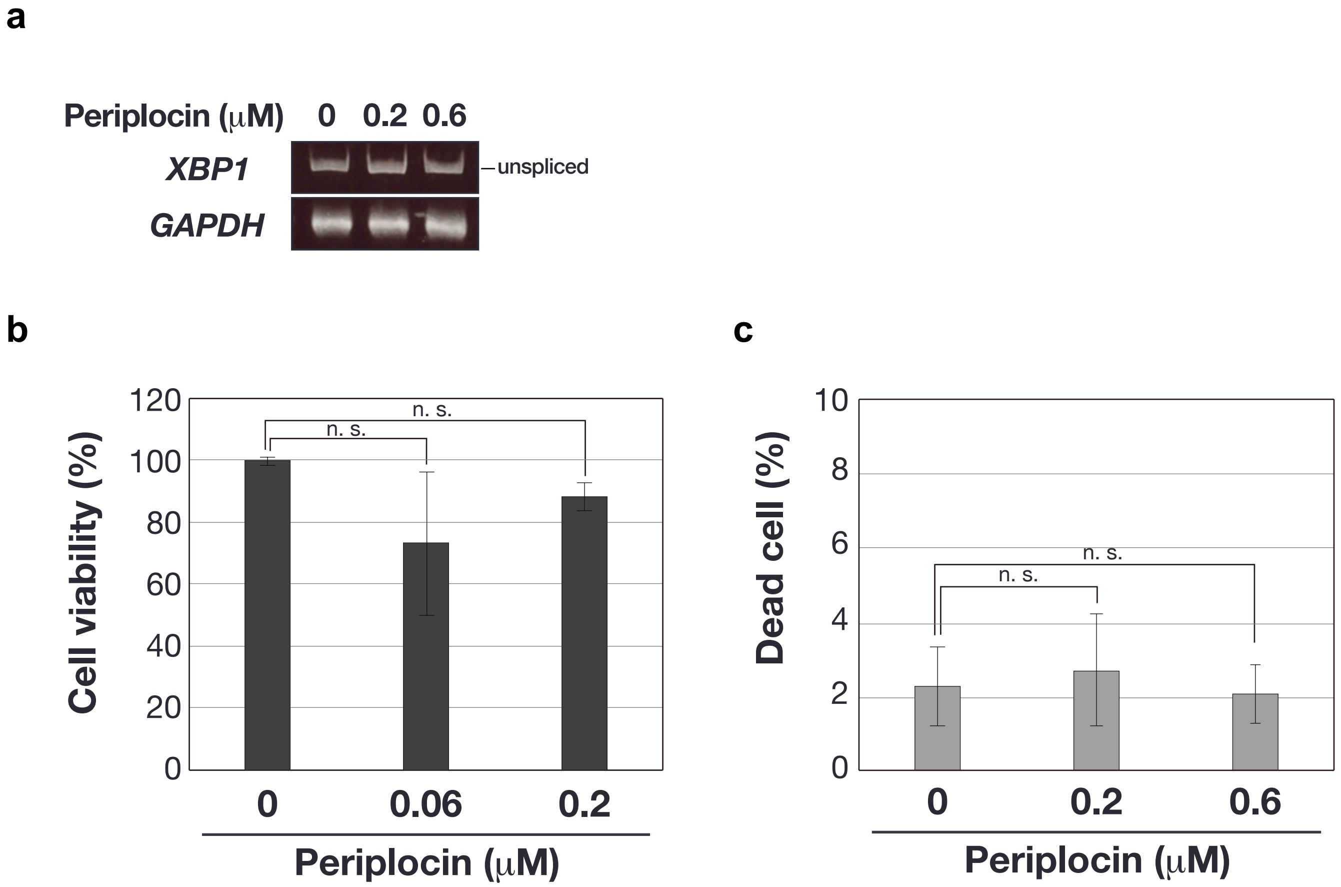


**Supplementary Figure S6** An evaluation for bioactivity of periplocin on normal fibroblast.
(**a**) TIG-1 cells were incubated with the indicated doses of periplocin for 24 h. The expression of each gene was assessed by RT-PCR. Unspliced *XBP1* mRNA is indicated. *GAPDH* was used as the loading control. (**b**) TIG-1 cells were incubated with the indicated doses of periplocin for 24 h. Cell viability was measured by the WST-8 cell proliferation assay. Results are shown as the mean ± S.D. ($n$ = 3 biological replicates). Significant differences are indicated as $^{**}p < 0.05$, assessed by one-way ANOVA with Dunnett's post-test; n. s., not significant. (**c**) TIG-1 cells were incubated with the indicated doses of periplocin for 24 h. The percentage of dead cells was measured by trypan blue staining. Results are shown as mean ± S.D. ($n$ = 3 biological replicates). Significant differences are indicated as $^{**}p < 0.05$, assessed by one-way ANOVA with Dunnett's post-test; n. s., not significant.

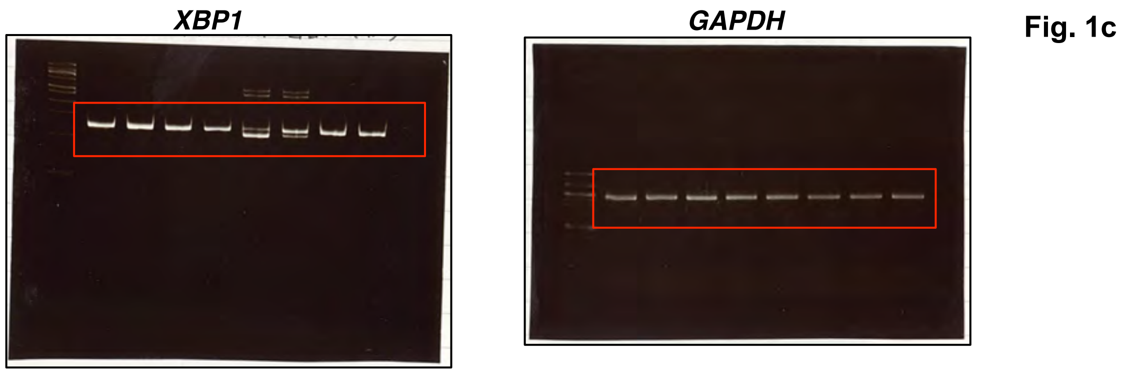


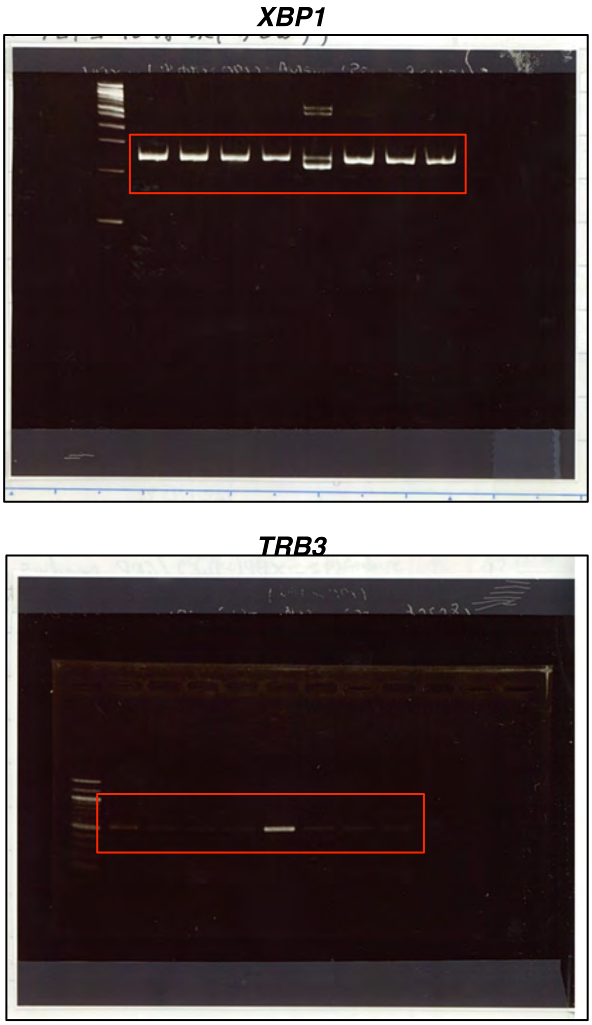


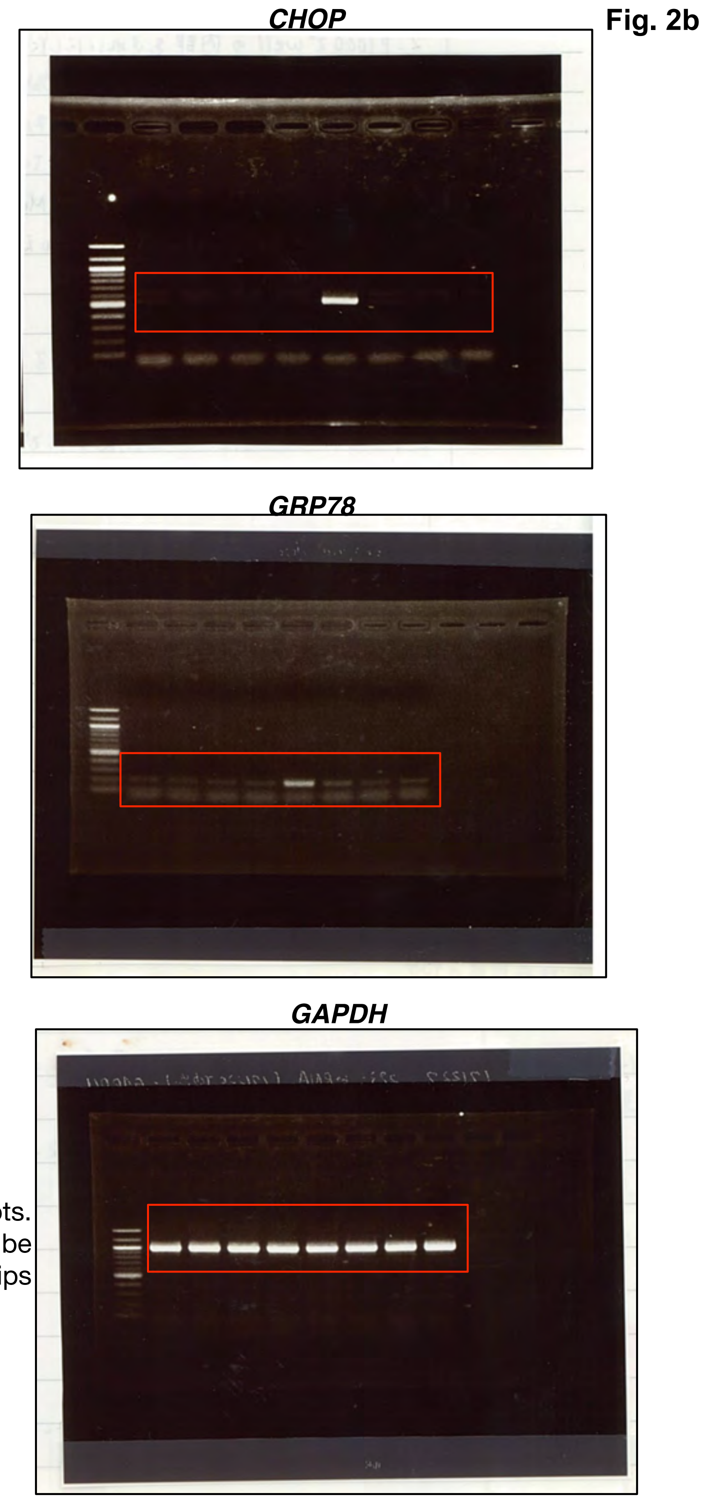


**Supplementary Figure S7** Uncropped data of gels/blots. Red boxes show the parts used in the figures. Please be noted that some membranes were cut into multiple strips prior to immunoblotting to detect multiple antigens.

## Supplementary Figure S7

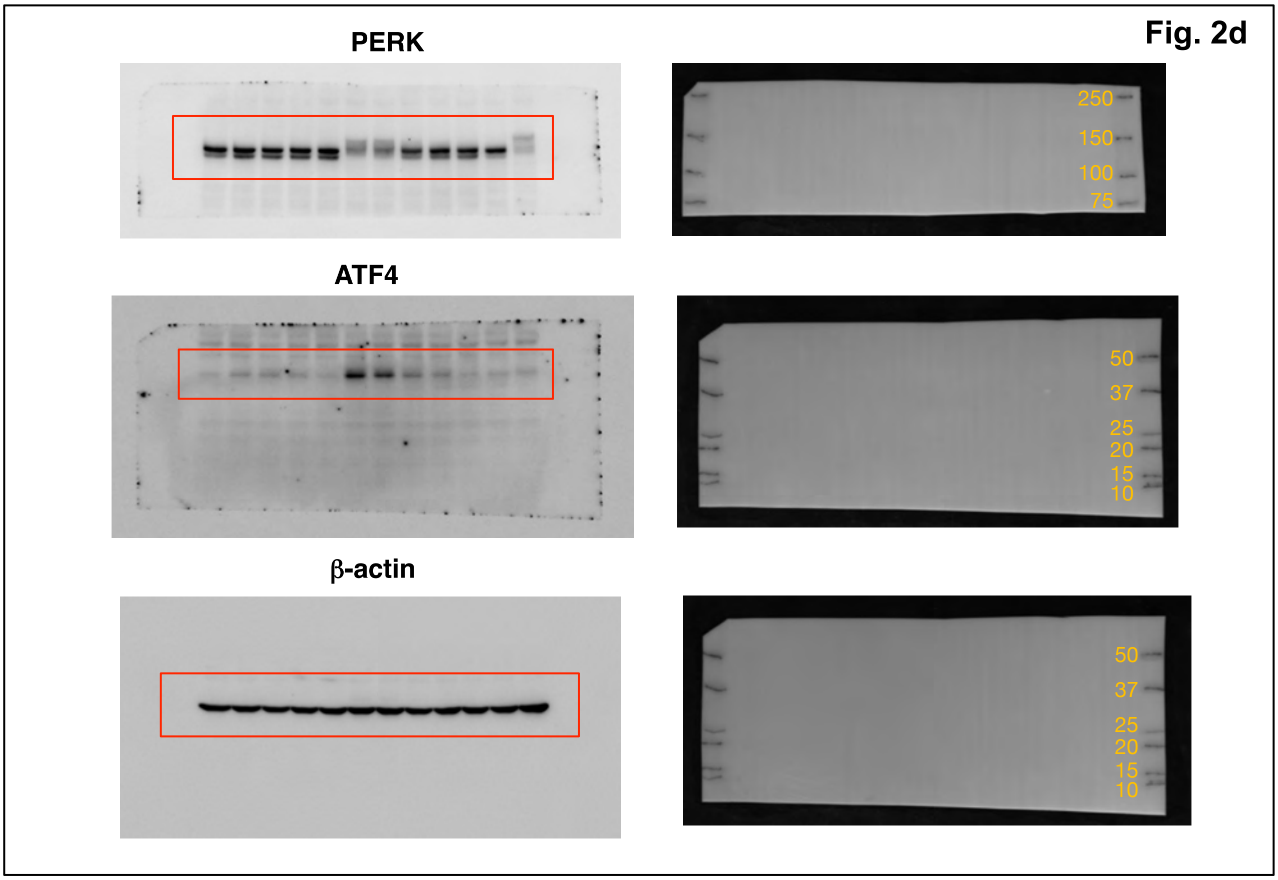


## Replicates for Fig. 2d

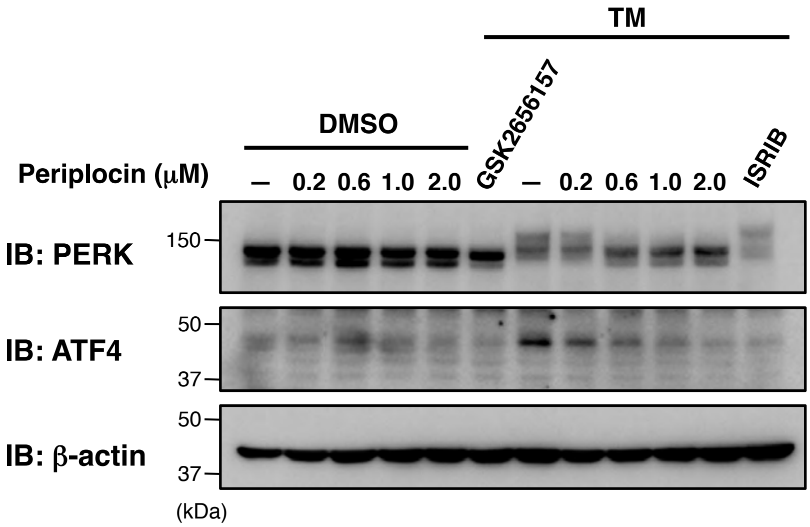


**Supplementary Fig. S7 continued**

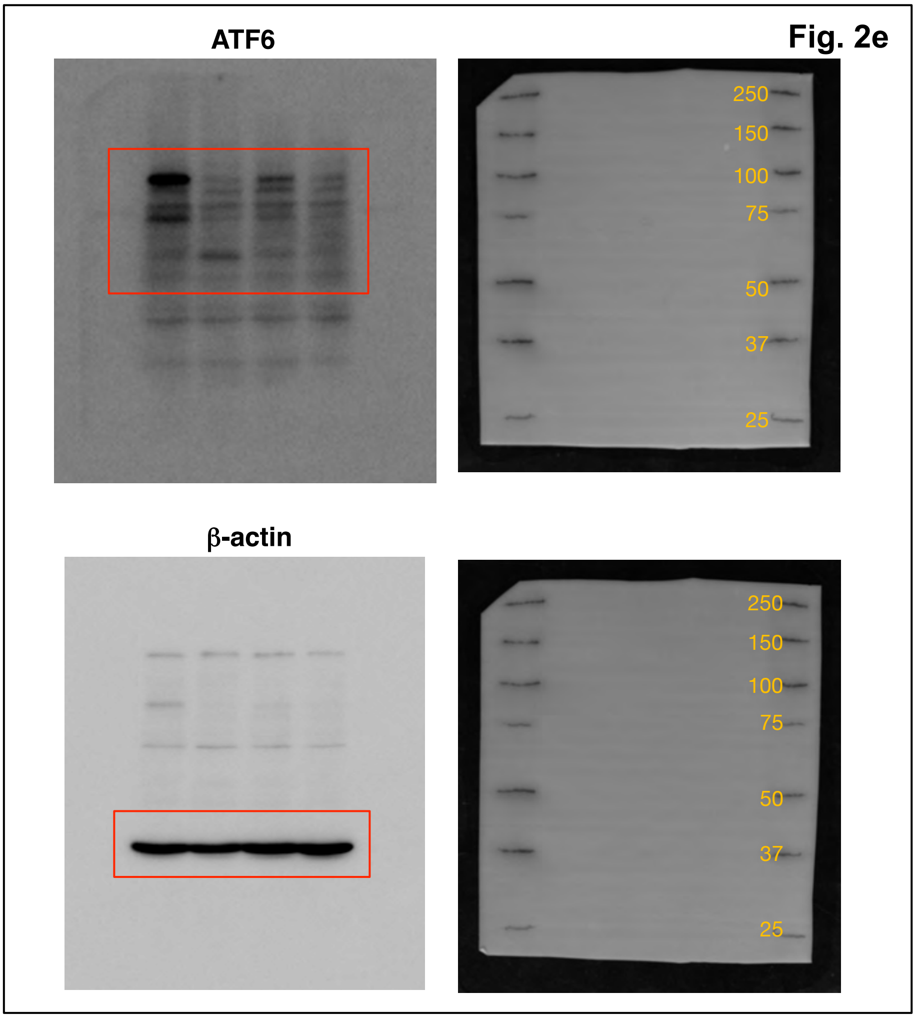


## Replicates for Fig. 2e

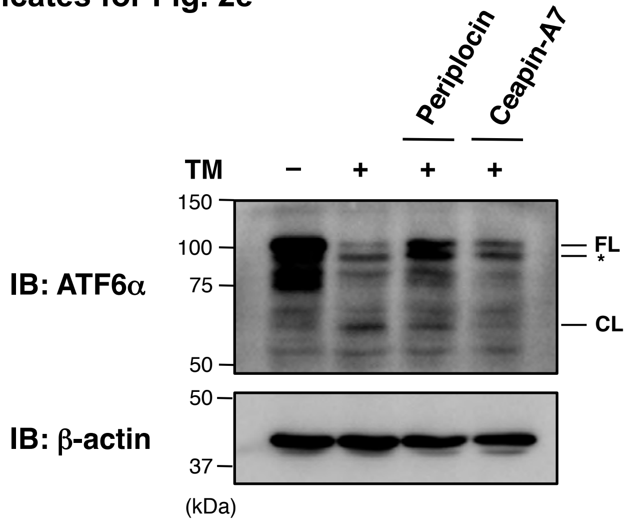


**Supplementary Fig. S7 continued**

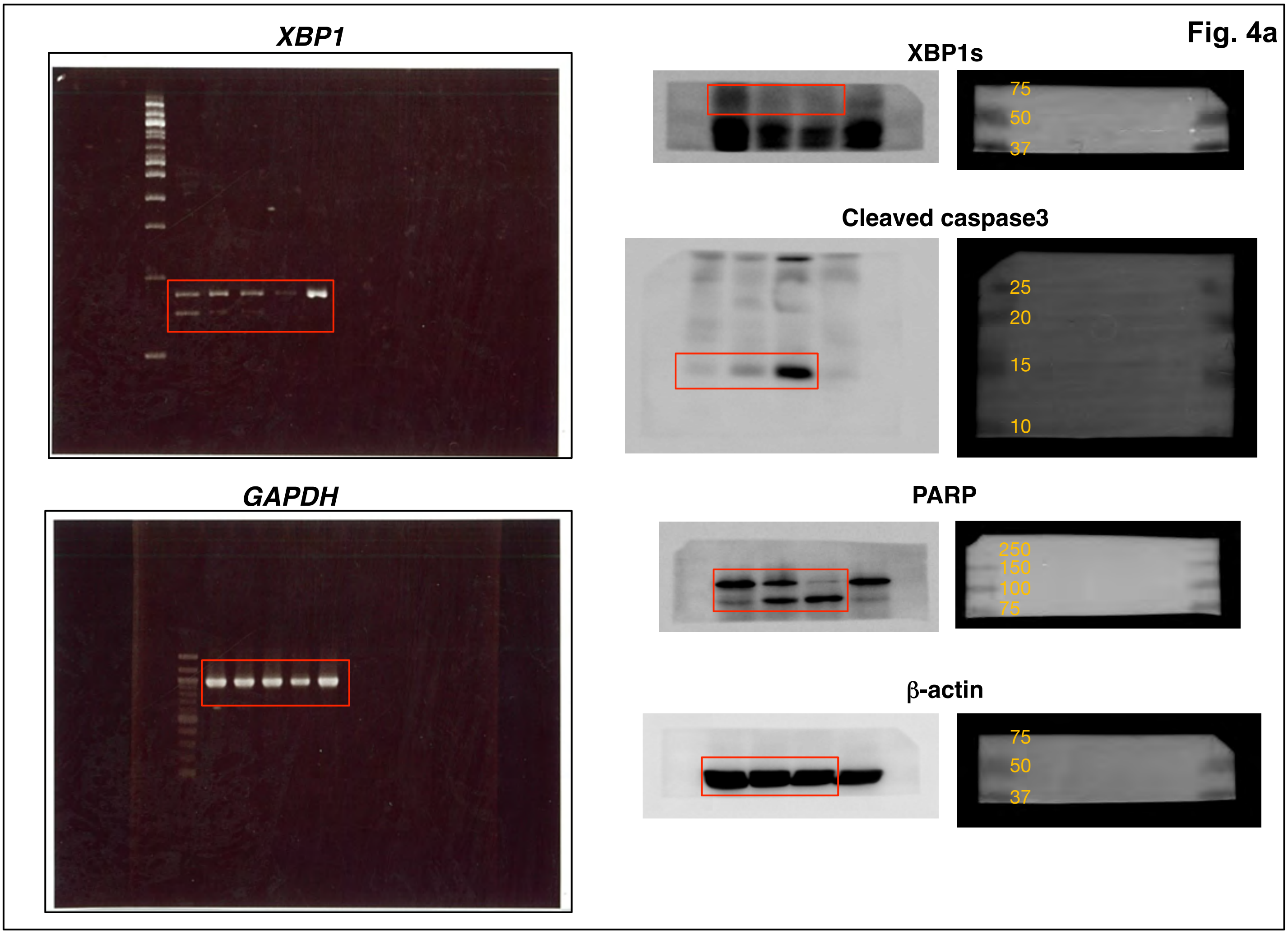


Replicates for Fig. 4a

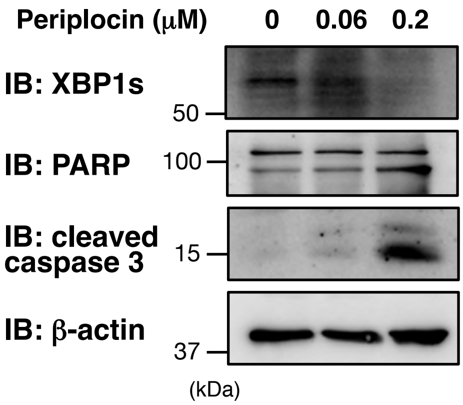


Supplementary Fig. S7 continued

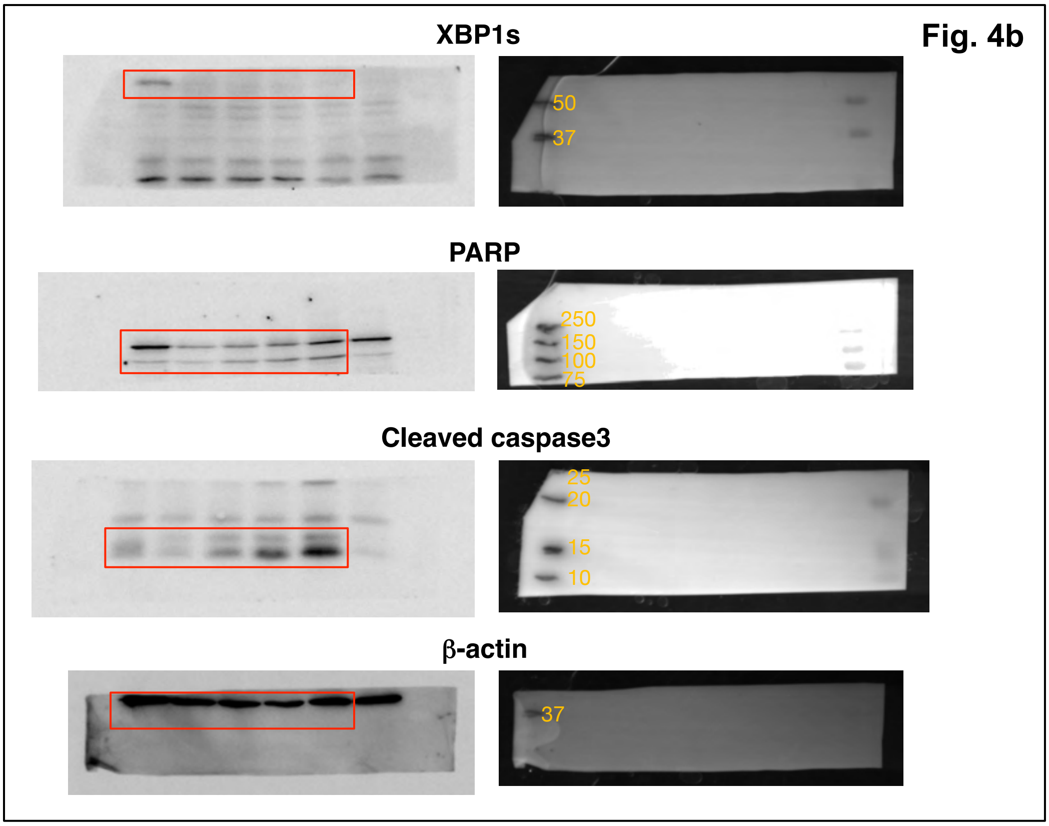


## Replicates for Fig. 4b

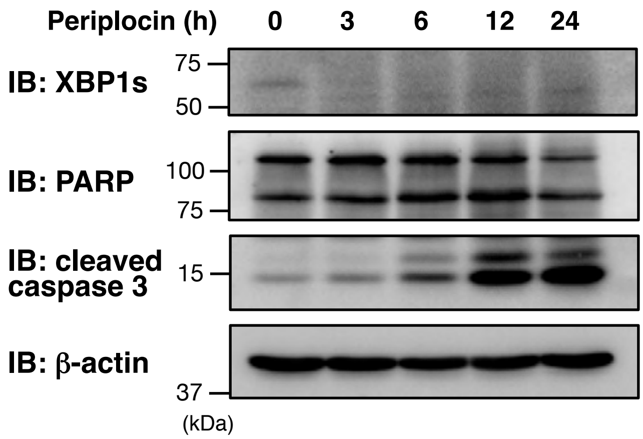


**Supplementary Fig. S7 continued**

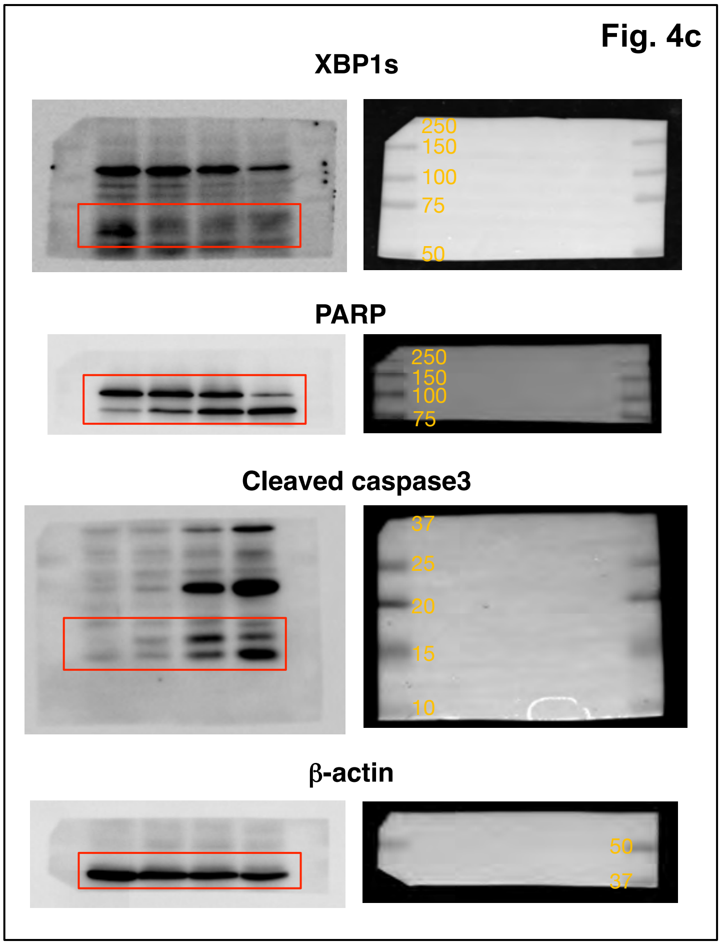


## Replicates for Fig. 4c

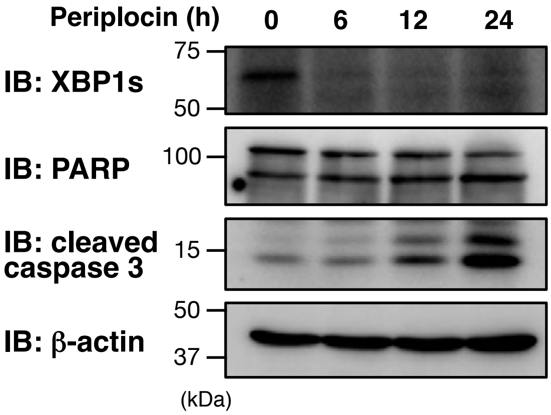


**Supplementary Fig. S7 continued**

## Supplementary Fig. 5b

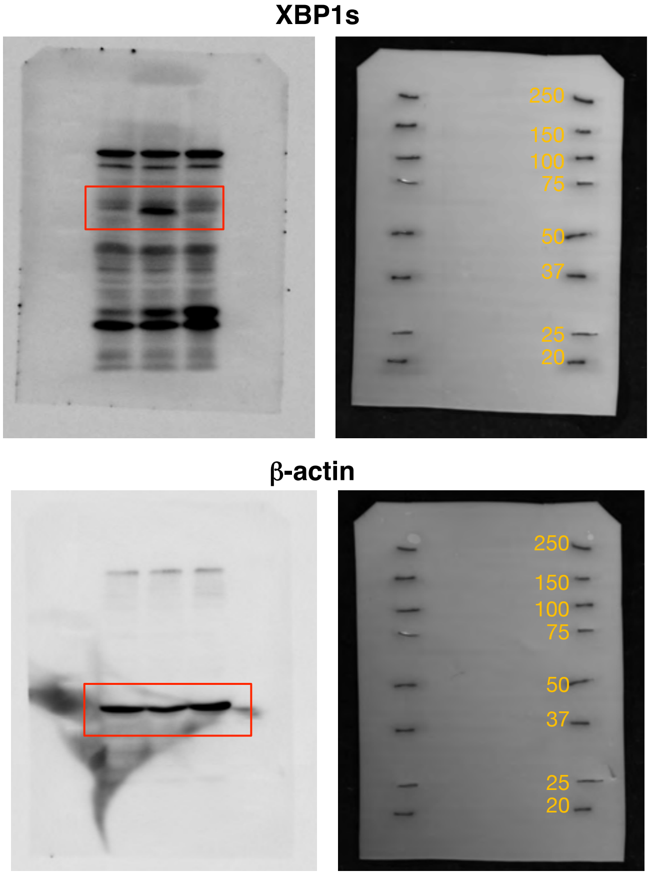


## Supplementary Fig. 6a

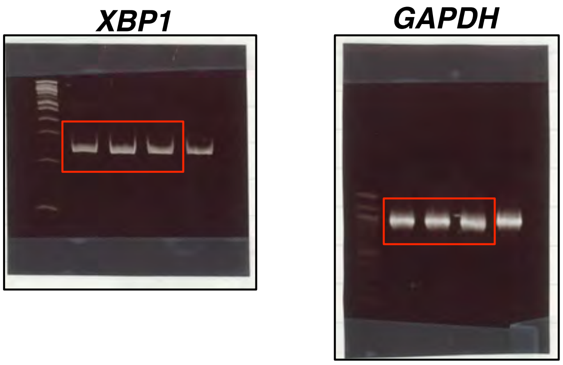


## Supplementary Fig. 5a

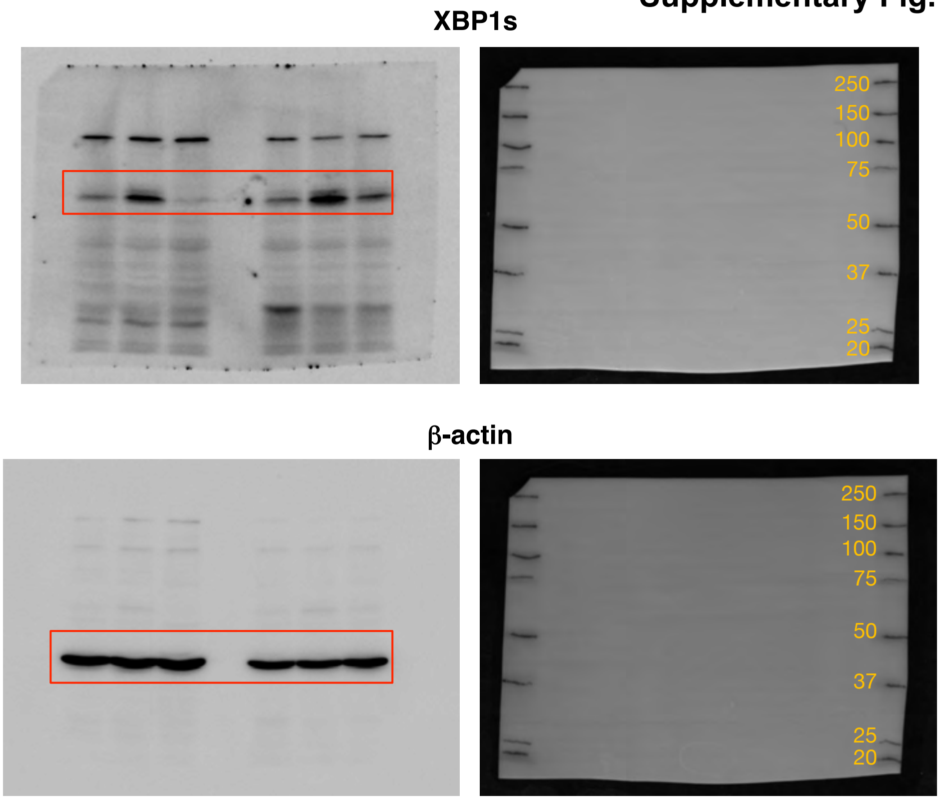


**Supplementary Fig. S7 continued**
